# Border Crossing and the Logics of Space: A Case Study in Pro-Environmental Practices

**DOI:** 10.3389/fpsyg.2018.02096

**Published:** 2018-11-01

**Authors:** David Uzzell, Nora Räthzel

**Affiliations:** ^1^School of Psychology, University of Surrey, Guildford, United Kingdom; ^2^Department of Sociology, Umeå University, Umeå, Sweden

**Keywords:** border crossing, spillover, environmental practices, health and safety, institutional logics, home and work, life histories, behavior change

## Abstract

We investigate whether and how workers in a transnational oil corporation carry practices, meanings, and identities between the places of work and home, focusing on environmental and health and safety practices, in order to understand the larger question, how can environmentally relevant practices be generalized in society at large? Our theoretical starting point is that societal institutions function according to different logics (Thornton et al., 2012) and the borders (Clark, 2000) between these institutions create affordances and constraints on the transfer of practices between these places. By connecting their theoretical ideas, we suggest that these provide an alternative critique and explanatory account of the transfer of environmental practices between home and work than a “spillover” approach. We employ life history interviews to explore the development and complexity of the causes, justifications, and legitimations of people’s actions, social relationships, and the structural constraints which govern relationships between these spaces. While Clark’s concepts of permeable, strong, or blended borders are useful heuristic tools, people may simultaneously strengthen, transgress, or blend the borders between work and home in terms of practices, meanings, identities, or institutional logics. Individuals have to be understood as creators of the border crossing process, which is why their life histories and the ways in which their identities and their attachments to places (i.e., institutions) are shaped by the logics of these places are important. For environmental practices to travel from work to home, they need to become embedded in a company culture that allows their integration into workers’ identities.

## Introduction

Our interest in the ways in which individuals might take practices, identities, and meanings from one place to another is rooted in our concern for environmental change, which requires a transformation of the way we produce and consume. Thus, whether and how people might take practices from sites of production (work) to sites of consumption (home) is crucial for understanding how such a transformation might occur. This research might seem to fall under the heading of spillover, but most studies of spillover focus on transfer across domains [e.g., waste behaviors and energy conservation (Thøgersen and Ölander, 2003; Poortinga et al., 2013; Thomas et al., 2016]. Our focus is on place and the conditions for transfer may be very different. “Spillover,” “catalyst,” or “wedge” approaches are methodologically empathetic with neoliberal government market-led strategies (Defra, 2008) which require neither legislative levers nor structural transformation that challenge consumer sovereignty, and rely on individuals’ own preferences and decisions in the context of influencing “choice architectures” (Thaler and Sunstein, 2008), social marketing (McKenzie-Mohr, 2000; Cialdini, 2003), or encouraging particular environmental identities (Stryker and Burke, 2000; Nigbur et al., 2010; Whitmarsh and O’Neill, 2010).

There is little doubt that environmental psychologists have been at the forefront of research seeking to understand the drivers and constraints on individuals’ environmental behaviors, drawing particularly on theories from social psychology, e.g., attitudes, social norms, and behavior change (Clayton et al., 2015). But in recent years, it has been recognized that a more particular contribution that environmental psychology might make is to draw on research which has focused on the importance of place in people’s lives through concepts such as place identity and place attachment, and explore how these are functional for people’s environmental behaviors and practices (Uzzell et al., 2002; Clayton et al., 2016). Building upon this development, we sought to find an alternative approach to “spillover” that provides a more nuanced understanding of the transfer of meanings and practices across different places, and moves away from the kind of individualistic approaches described above. We thus posed the question, under what conditions are environmentally significant practices carried from home to the workplace and *vice versa*?

In order to answer our question, we recognized the need for a concept of place which incorporates the specificities of home and work. The first author is an environmental psychologist whose work over many years has explored how people develop place attachments and place identities and how such responses may be functional for pro-environmental behavior and the support of environmental practices. The second author comes from sociology where the interest is on the societal structure of a place: what is the societal goal of place, what are the societal rules governing the actions at this place, and how do these shape the ways in which people act and think about places? To bring together the approach of environmental psychology and sociology, we draw on two theoretical approaches to make sense of our material: Clark’s (2000) theory of border crossing, which centers on individuals as conscious actors, and the theory of institutional logics (Friedland and Alford, 1991; Thornton et al., 2012) which analyses the societal structures of places. In this theoretical framework, the places of home and work are institutions, socially created places, with specific societal goals and specific rules and regulations (logics) governing what kind of practices can (and must) take place there and which ones are “out of place,” need to be avoided. In the following, we will use the terms “place” and “institution” interchangeably.

The paper is organized as follows: first, we elaborate on our usage of Clark’s border crossing and the institutional logic perspective; second, we describe our methodological approach. The third part comprises the analyses of our material: a diachronic, in-depth analysis of two case studies which exemplify the multidimensional and contradictory relationships our protagonists developed between home and work against the background of their life-histories, and a synchronic analysis which offers an investigation of the breadth of border crossing practices. Fourth, we conclude with a suggestion to combine border crossing with the institutional logic perspective, creating a perspective that includes process (border crossing), structure (institutional logics), and the individual as the actor.

## Theoretical Framework

The transfer of environmental practices between work and home has typically been examined through the concept of spillover, but this has been shown to have many shortcomings and mixed results (Grzywacz and Marks, 2000; Thøgersen and Crompton, 2009; Austin et al., 2011; Littleford et al., 2014; Truelove et al., 2014). One particular failing is the lack of the concept of actors, of individuals having emotions, making sense of their worlds and deciding to take practices from one space to another. These problems have been compounded by the use of measures of statistical association, and inadequate theoretical attention being paid to *how* it works when it does (Thøgersen and Crompton, 2009; Austin et al., 2011). Clark’s (2000) concept of “border crossing” overcomes this shortcoming by formulating questions from the point of view of the individuals: “People are border-crossers who make daily transitions between two worlds – the world of work and the world of family. People shape these worlds, mold the borders between them, and determine the border-crosser’s relationship to that world and its members. Though people shape their environments, they are, in turn, shaped by them” (ibid.: p. 748). Clark was concerned with issues of work–home balance, but we felt that the concept of border crossing had wider utility and might be used to explain how individuals take practices from work to home and *vice versa*. In this paper, we focus principally on three key characteristics of Clark’s borders – permeability, blending, and strength. Permeability is the perviousness of a space and the degree to which practices and behaviors from one place/institution are able to enter another. Strength is the degree of resistance as one moves from one place to another. Spatial and temporal blending occurs when there is a high level of border permeability creating a “no-man’s land,” which is neither exclusively home nor work, for example, a “spare” bedroom converted into an office or when a dining room table is used in the evening for office work that has been brought home.

The origins of border crossing theory lie partly in the work of Kurt Lewin (Lewin and Cartwright, 1952) and his concept of “life space.” Lewin believed a life space includes attitudes, memories, and motivations which are set within environmental and situational contexts or “regions” that have borders which are subject to different degrees of permeability. For Lewin (1948), the boundaries between life space regions have two important qualities, sharpness and rigidity. Clark (2000) took this idea and its later formulations and suggested there are four elements to the theory, (a) two spaces, in this case work and home, (b) the borders between the two, (c) the agent, i.e., the border-crosser, and (d) the border-keepers and other domain managers. She explained the differences between home and work as follows: “Differences between work and home can be classified in two different ways: differences in valued ends and differences in valued means (…)” (ibid.: p. 753). Clark found that work is predominantly valued because it provides an income and gives “a sense of accomplishment”, while home life satisfied the ends of attaining close relationships and personal happiness” (ibid.) What is lacking in this explanation is a sense of the affordances and constraints of the two places which could explain why people give different values to different places, for instance, by analyzing them as societal institutions. What is needed is a language that enables us to understand the institutional structures and settings in which individuals operate, and within which people value different places differently.

The theories of “institutional logics” provide such a language. Its first promoters were Friedland and Alford (1991), who drew on Mary Douglas’s anthropological insights (Logue et al., 2016) as a means of examining the interrelationships between individuals, organizations, and society, in particular the idea that everyday practices are place-related such that actions are made meaningful in the context of social relations within different institutionalized structures. They contended that, “… institutions shape individual preferences and organizational interests as well as the repertoire of behaviors by which they may attain them. These institutions are potentially contradictory and hence make multiple logics available to individuals and organizations” (ibid.: p. 232). In their book on the “Institutional Logic Perspective,” Thornton et al. (2012) developed the theory further and created an authoritative framework for research on the “culture, structure, and process of institutions.” The theory aims to understand institutional logics from within their respective practices relating them to societal logics at large only in as far as they can be observed at specific conjunctures. One of their examples is the effect of the changing power of market logics on companies (ibid.: p. 77). While we find the idea of institutional logics useful we would like to suggest a different way of creating institutional categories. For instance, Thornton et al. describe some family logics with the same concepts used for the functioning of companies: “increasing,” “capitalism,” “status.” (ibid.: p. 73). In our view, it is possible to differentiate between more or less powerful logics which may lead to the infiltration of dominant logics from one institution setting to another (a capitalist logic entering the family logic). However, such processes cannot be analyzed critically if their results are already taken for granted by the usage of the same categorical logics for different institutions. Therefore, we suggest to define the logics of an institution according to the role(s) it plays for the reproduction of society. These would be defined as the essential logics, while contingent logics would be those which help to realize this role but would differ according to place and political conjunctures.

The role of production consists in producing the means for life, while the role of families consists in producing life itself. These general and basic definitions offer a starting point from which to formulate the essential logics of an institution. More concretely, in capitalist market societies corporations need to produce a product that appears useful to their customers and that creates a profit to satisfy shareholders. The logic of profit and the logic of use-value are thus the essential logics of corporations. For families to fulfill their role of creating the next generation and providing a space where people can regenerate to continue to work productively the logic of care is essential. Interestingly, the logic of care does not figure at all in the definition of the institutional system that Thornton et al. (2012, p. 73) provide. Essential logics are those which an institution cannot disregard without endangering its existence, while contingent logics change according to place and time and can be realized or not.

Given the different logics under which life in the workplace and in the place of home are lived, it makes sense to talk about the transfer of behaviors and practices between work and home in terms of border-crossing. Actions that are functional in the workplace can be dysfunctional at home and *vice versa*. For example, most parents would not want to put their child to bed evaluating the process in terms of “time/effort input and output.” If they have to, due to conflicting needs, they may feel guilty. This is not to say that the institutions of families and companies cannot share certain logics. We can find the contingent logic of care in a company devoted to environmental protection and the contingent logic of cost efficiency in families needing to make ends meet. Some workplaces are designed for the home/work border to become fuzzy (Enigma, 2016), e.g., break-out spaces where the provision of armchairs and coffee tables may encourage a relaxed environment, implying that work and life are indistinguishable (Michel, 2011). However, this serves to stress the difference between the two institutions, since the aim is to make employees feel more “at home” assuming this will make them feel happier and thus work better.

In addition to combining the theoretical approaches of border crossing and institutional logics, we decided that life-history interviews would be the most appropriate method to answer our questions (Denzin and Lincoln, 2003; Daiute and Lightfoot, 2004; Portelli, 2015). They capture the complexity of the family/work institutional context over time. Life-histories allowed us to explore the processes and conditions under which people engage in, or refrain from carrying practices across work-home borders. They help us to understand the contradictory attitudes that people can hold in the pursuit of socially desirable goals, as well as the causes, justifications and legitimations for their practices.

To summarize, if we are to understand more fully the processes which govern the transfer of actions across the work/home border, there are at least three issues which need to be addressed. First, we need a framework that articulates the transfer process, which we find in combining Clark’s theory of border crossing and a revised version of the institutional logics perspective. Second, there is a need to understand the interactions between and the complexity of the psychological processes, social relationships, attachments, and identities which are shaped by structural constraints and affordances. This requires us to put the individual at the center of the process. Third, in order to do this, we need to use a methodology that captures the developmental dynamics of changed practices and their transference across institutional spaces, which we find in using life-history interviews.

## Crossing Borders: Context and Methods of Investigation

The research reported here was part of a larger international study which examined the lifestyles, working practices, and home/work spaces of workers in seven countries and a variety of workplaces. This case study analyses the life of on- and offshore oil workers and managers in a major transnational corporation, GlobalOil^1^ operating in the North Sea. The separation of home and work is more extreme in the oil industry than in many others and therefore it highlights some of the consequences of the growing economic and lifestyle trends of hypermobility that lead to physiological, psychological, emotional, and social costs (Cohen and Gössling, 2015). GlobalOil was interested in the research as they had put into place the environmental program “Sustainable HomeWorks” (name changed) to encourage employees to engage in environmentally sustainable behaviors in the office and at home.

Our interviews were introduced by an explanation of the project followed by the request for individuals to tell us their life history. We only asked questions for clarification related to what interviewees told us, because we wanted them to determine the content of their life-stories. If issues we were interested in were not mentioned we asked about them at the end of the interview. Some interviews (with senior managers at GlobalOil headquarters) were semi-structured informative interviews, focusing on the problems and possibilities of implementing environmental practices in the company. Our contract with GlobalOil required that the interviewees (except for HQ managers) were selected by the HR Manager of GlobalOil; the reason given was that it was necessary for logistical reasons (e.g., availability when flying out to oil platforms). Unavoidably, this gave HR some degree of control over selection. In addition, we used a snowballing method to recruit more oil workers employed by other companies but working under similar conditions. Interviews were conducted onshore either before or after deployment to a platform. On acceptance, all interviewees were provided with an information sheet explaining the purpose, procedure, and ethical aspects of the project and a consent form to confirm their willingness to participate. Twenty-five interviews were conducted between February and June 2012 in London and Aberdeen lasting between 1 and 2½ h, resulting in + 60 h of interviews (Table 1). In Aberdeen, 11 off-shore workers (one female) and seven on-shore staff (two female) holding management positions were interviewed. In London, four senior staff were interviewed (one female)^2^. All interviews were recorded and professionally transcribed. We consulted published GlobalOil reports on their environmental record, as well as documents of regulatory government bodies, in order to understand the company’s public representation of their environmental practices. The research received a favorable ethical opinion by the University of Surrey Ethics Committee^3^. The names of interviewees are pseudonyms to avoid personal identification.

**Table 1:** GlobalOil interviewees.

Scott Adams	Offshore technician; later onshore office	M	GlobalOil	Aberdeen and North Sea
Kia Alani	Offshore and onshore engineer	F	GlobalOil	Aberdeen and North Sea
Robin Banks	Offshore operator	M	GlobalOil	North Sea
Tony Sarkus	Wiring technician (offshore)	M	GlobalOil subcontractor	Aberdeen and North Sea
Will Brennan	Diver (offshore)	M	GlobalOil subcontractor	North Sea
Kevin Dale	Offshore operator	M	GlobalOil subcontractor	North Sea
Conor Davies	Mechanical engineer	M	GlobalOil subcontractor	Aberdeen and North Sea
Paul Evans	Mechanical engineer	M	GlobalOil	Aberdeen and North Sea
Andy Harper	Offshore operations supervisor	M	GlobalOil	North Sea
Gary Holmes	Trade union official	M	Oil Industry Trade Union	Aberdeen
Buck Jones	Project manager	M	GlobalOil	Aberdeen and North Sea
Frank McKeen	Operations supervisor	M	GlobalOil	North Sea
Rona Mills	Finance manager	F	GlobalOil	Aberdeen
Steve Morris	Technician	M	GlobalOil subcontractor	North Sea
Anne Pedersen	Senior manager	F	GlobalOil	Aberdeen
Jim Roberts	Senior manager	M	GlobalOil	London
Brian Smith	Senior manager	M	GlobalOil	London
Nick Stevens	Operations manager	M	GlobalOil	Aberdeen
Emily Stevenson	Senior manager	F	GlobalOil	London
Matt Thompson	Environmental manager	M	GlobalOil	Aberdeen and North Sea
Luc Vermeeren	Project manager	M	GlobalOil	Aberdeen
Mike Wellwood	HR manager	M	GlobalOil	Aberdeen
Chris Williams	Environmental manager	M	GlobalOil	Aberdeen
Philip Woods	Senior manager	M	GlobalOil	London
Dave Wright	Offshore installation manager	M	GlobalOil	North Sea

### Analytical Strategy

Our aim was to undertake a nuanced analysis exploring what border crossing might mean in respect of the transfer of practices, meanings, and identities from one domain to another, rather than in relation to work/family balance which was the objective of many studies which draw on the concept (Geurts and Demerouti, 2003; Shumate and Fulk, 2004). Because life history interviews covered many aspects of the individuals’ formation, career development, and domestic and working lives, we approached the coding and data analysis with specific questions in mind (Braun and Clarke, 2006). We were interested in (a) the rationale and legitimations interviewees provided for their decisions, (b) the institutional logics by which they were framed, and (c) what kind of practices, meanings, and identities were taken from one domain to another. Having identified these elements, we assessed whether Clark’s categories of permeability, blending, and strength provided a suitable classificatory framework for the analysis. It was not, however, a question of fitting the material into Clark’s framework as a form of confirmatory analysis. Quite the opposite, we were concerned to identify under what conditions border crossing occurred, and what conditions led to its resistance where there were contradictions with or divergences from the model that border crossing and the institutional logic perspective present. This will become clear in our diachronic analysis.

Thematic analysis was considered to be the most appropriate for the identification of “repeated patterns of meaning” regarding the transfer of practices between home/work (Braun and Clarke, 2006). The transcribed interviews were imported into MAXQDA11. They were coded taking several steps: first, we coded all instances where interviewees talked about a transfer of practices, meanings, or identities from home to work or *vice versa*. Second, we coded instances where we found that similar practices, meanings, or identities reported by interviewees appeared in descriptions of their work and their home practices. Third, we used Clark’s generic concepts (e.g., permeability, strong borders) to create sub-codes. While our coding sought to draw on Clark’s categorical concepts, we were open to different formulations of border-crossing than discussed by Clark as will become apparent in our analysis through the coding of the semantic and latent content. Finally, we created a further sub-group of coded instances by coding who supported or resisted border crossing (i.e., the worker, members of his/her family, and friends).

By setting the interviews in their situational context, we sought to explicate and give meaning to individual, institutional, and societal drivers. Our analysis not only seeks to describe cross-border movement of practices, meanings, and identities, but also the underlying assumptions and drivers of these movements. It is necessary to look at individual motivations to explain human behavior, but in common with the position we take in much of our work on behavior change, we are concerned not to ignore the affordances, constraints, and logics of the places in which people live out their everyday lives.

Because we considered Clark’s concepts to be sufficiently general to allow a diversity of theoretical explanations, our approach was essentially theoretically inductive. In the synchronic analysis, themes and sub-themes were developed by collecting accounts which were related, and these were discussed in order to confirm their validity and to ensure that their interpretation was convincing and defensible. It was an important part of the analytical strategy that we were sensitive to themes which were identified inductively.

Life on an oil platform with its hostile working environment and crowded living conditions bears little comparison with the working experiences of most people. Thus, it might be thought of as an inappropriate case study for understanding the “everyday” experiences of workers and their relationships with their workplace, family and wider society. However, oil workers’ lifestyles bring into sharp relief many of the issues affecting home/work relations and the barriers to change which exist in other contexts as well. As Flyvbjerg has argued, “extreme cases reveal(s) more information because they activate more actors and more basic mechanisms in the situation studied. In addition, from both an understanding oriented and an action-oriented perspective, it is often more important to clarify the deeper causes behind a given problem and its consequences than to describe the symptoms of the problem and how frequently they occur” (Flyvbjerg, 2006, p. 229). Because most workers live in a societal and family context (Morrice et al., 1985; Sutherland and Flin, 1989), these contrasting structures will always create different values and issues. A workplace is not only a place of work but also a place where social relations, friendships, and enmities are created. How this happens becomes more visible when the workplace becomes a “home from home” for a longer period of time as in the case of workers on an oil platform.

Qualitative data are sometimes criticized for not permitting generalizability. But as Tsang (2014) and Eisenhardt and Graebner (2007) have found, case studies are increasingly used for theory development, not only because they are sensitive to context and the conditions under which phenomena may occur (perhaps in one setting but not another as is the case in this study) but also because they “allow researchers to tease out ever-deepening layers of reality in the search for mechanisms and influential contingencies” (Tsang, 2014), and to gain insight into the factors linking cause and effect (Gerring, 2007), which may have policy lessons for home/work relations applicable across the economy.

To examine such layers, we undertook both a diachronic and a synchronic analysis of our material. In the diachronic analysis, we focus on two off-shore workers and how living, as one worker called it, “two lives,” impacts on them, their friends, families, and their environmental and safety practices. The focus on two examples aims at an in-depth analysis of the complexity and contradictions of home–work relationships and introduces the individual and their life-story as a mediator of these relationships. The synchronic analysis in turn, presents a broader variety of home–work relationships to understand which kinds of practices make their way across the borders.

## Diachronic Analysis: the Ambiguities of Border Crossings and the Power of Company Logics

For the diachronic analysis, we have chosen two individuals who are both “extreme” when compared to the majority of “normal” working conditions and “paradigmatic” (Flyvbjerg, 2006). They are paradigmatic because all offshore workers – including Kevin Dale and Andy Harper who are the key protagonists in this diachronic analysis -talked about their offshore life becoming a home from home. But they differed in terms of the ways in which the border was managed between their two homes. This was due to their different life histories and stages of life.

After a short introduction to the workers’ background, we present their relationship to and identification with their company. Identification is important for the ability of individuals to manage the work–home border successfully (Clark, 2000) and to comply with the role of an institution (Thornton et al., 2012). We then analyze the ways in which our protagonists describe and define their working places and their homes, manage borders, carry practices and identities from one place to another, and relate to the logics of company and home.

### Kevin Dale – Strong and Permeable Borders

Kevin Dale, an offshore subcontractor aged 23, had trained with several oil companies from the age of 16 after leaving school. Dale is single and lives on his own. He is a Process Technician and responsible for quality and environmental protection:

And I’m shouted to control, maintain or modify anything that’s happened on the plant, to meet the standards for export so that we’re keeping everybody happy. Avoiding unnecessary shutdowns where possible, unnecessary flaring where possible, or any kind of discharge to the sea where possible.

Meeting standards for export is necessary, while avoiding actions harmful to the environment are conditioned on their possibility. Dale identifies with the environmental record of his company, describing it as “*very good*” since they have a policy of zero discharge into the sea whereas 20 years before, “*it would just be a ring of a slick every way*.” When he describes his work offshore its contradictions become evident:

… my work is quite interesting. (…) I’m used to it now. I’ve done it for so long, I wouldn’t really know how to do a nine-till-five office job. (…). But yeah, you just tend to work. Work, gym, sleep. Work, gym, sleep. And then that’s you ready for home! And you try not to count down the days and, you know, count your life away a wee bit. But that tends to be what everybody does. You’re looking forward to that day you’re going home. Then you come home and it’s the best job in the world! You think it was the worst job when you went out there; when you come home it’s the best job in the world!

The change between an intense time at work and an intense time at home creates a solid barrier between the two. Work itself is rewarding, but the employment conditions of having to work 12 h a day 2 weeks in a row on what appears like a monotonous routine makes his job appear as “the worst.” The best part of the job is being able not to do it, to enjoy 3 weeks onshore without any work commitments. At the platform itself, the social relations, the familiarity with co-workers bring a comfort and a compensation for the hard work routine:

And I’ve worked with the same group of about a dozen guys now for the last couple of years, so we’re very friendly. (…) we’re all very close and we get on well together. (…) it’s nice to go out there and know what you’re getting, knowing the people, having your own same cabin. (…)You know the gym, you know what the food’s like, and it’s just easier. (…) It’s like a home from home.

It is his relations with friends, which permeate the borders between work and home:

And in my spare time I like to – I’ve got a very close, good group of friends. We like to go on weekends abroad. Quite often we – well, we go to Barcelona nearly every year. I’m going this weekend to watch the MotoGP motorbikes. And we often take trips down south … by plane – or by train at times.

While it does not seem that his friends at work and his friends outside work are the same, the culture of male bonding is described similarly for both spaces. However, talking about the masculine culture at the workplace which he describes as rough, he constructs a contrast between the two domains: “*I think you tend to mould yourself into that* [masculine culture at work]*, and then you come home and you’re a gentleman again for three weeks. And then you go back [laughs] to the regular way!*”

It is noteworthy that Kevin Dale sees what many would consider to be abnormal (i.e., living on an oil platform) as being “regular,” while being at home requires being different – indeed he talks about it like an actor playing a role – he is “a gentleman” for 3 weeks only. Given his description of practices outside work racing and watching motorbike races, it is not easily understandable why Dale uses the term “gentleman” to portray his behavior away from the rig. But it may be that home has a symbolic function for him – it is a place where you are well behaved because this is where you meet the opposite sex and you should not behave in a “blokey” way. It is also possible that being a gentleman is associated with freedom, freedom from work:

A lot of the offshore guys tend to like their motorbikes because there’s that freedom to go wherever they want when they’re home. …. I’m very passionate about cars. I’ve had quite a few nice cars since I started working. I’ve also got heavily into motorbikes (…) with a lot of road-riding.

On the one hand, there is a strong border between on- and off-shore, as one can read the freedom experienced with the bike as a compensation for the restriction of space and time, which rules life on the oil platform. On the other hand, there is permeability too. A masculine culture is lived in both places. While offshore and onshore life are contrasted in terms of constraining, exhausting and repetitive practices at work and the freedom to roam at home, Dale also recreates logics of home, close friendships, and a “place of his own” at the workplace. Equally, a masculine culture of enjoying machines and dangerous practices are taken from home to work and *vice versa*. It is impossible to say, which is the source and which is the effect. Space restrictions and routine at work are not of Dale’s making, but he makes the most of overcoming these conditions by fully realizing the freedom of space and time outside work. So far, the borders between work and home are simultaneously strong and permeable, but what about environmental awareness?

I’m very passionate about cars. So, carbon footprint-wise, I have a car, I have a van and I have a motorbike! So that’s me doing my bit!

When Dale talked about his environmental responsibilities at work, he seemed fully integrated into a logic of care for the environment. Avoiding discharges into the sea, flaring, and other environmental damages is part of his responsibilities. This stands in stark contrast to this sarcastic self-description of “doing his bit.” But one could argue that while Dale does not take his environmental concerns onto the helicopter when he goes onshore, he does identify with the tacit contradictory logic which guides his company, contributing to environmental destruction while simultaneously engaging in some practices of environmental protection. His identification with the environmental values he sees GlobalOil developing works as a kind of permission to take environmentally damaging practices (creating carbon emissions) from the workplace into his places of leisure. Dale was fond of motorbikes and cars before he started work at GlobalOil. Thus his life-history shows that the dimensions of the workplace with which people identify depends also on the priorities they set in their life outside work.

### Andy Harper: Dual Loyalties – One Identity

Andy Harper is an offshore supervisor in his mid 50s. He has been working at GlobalOil for over 10 years and in the oil and petrochemical industry prior to that. When we interviewed him, he was working on an assignment in Northeast England while his family home was in Scotland. He regretted the carbon footprint that his traveling between both places entailed. We do not know much about his life history because while we kept asking questions about his life, he wanted to talk predominantly about how environmental issues have accompanied him all his life, at home and at work. He talked about how his grandparents recycled everything and how his son has now come “*full circle*” growing his own vegetables and buying his clothes in second-hand shops. In this context, he explains how GlobalOil is today more environmentally aware than in the past:

I do see that people from the top, (…) seem to be doing the right thing. There’s environmental focal points and environmental reps – that’s their full-time job. (…) I personally think GlobalOil (…) are more environmentally friendly than some other companies.

However, 40 min later, Harper tells a story which contradicts this judgement:

You walk round the office there’s *£*100,000 cars in the carpark! (…) So as a boss, as a head of the company, a head of department, they will stand up in front of all the workforce and tell them, ‘You must put your cup in that bin, and you must put your paper towel in that bin,’ and then they walk outside and they get in a big four-by-four and they drive forty miles to home every day! So it’s this thing where we all like to think that we do a little bit for the environment, but how much do we really do? Because we like our lifestyle.

From talking about the contradictory behaviors of managers, Harper switches to seeing these as examples of general human weaknesses, ending his story with a statement about “us” and “our lifestyle.” A few minutes later however, big cars are explained, not just as a human weakness but as part of the company policy:

I think as a company it’s kind of encouraged. Because once you get to a certain level within the company, you get a company car allowance. (…) If somebody says to you, “You can have *£*500 a month allowance to spend on a car,” you’re not going to spend *£*200 a month – you’re going to spend *£*500.

In these stories, we can detect shifts between practices at work and practices at home. While GlobalOil has policies to reduce its environmental impact, the behavior of managers and staff outside work contradicts their environmental efforts at work. While car ownership can be defined as a practice outside work, the company crosses the borders between work and home by rewarding employment positions with the provision of company cars and car allowances. This border crossing follows the profit logic as higher positions are rewarded with higher allowances, enabling higher status at work to be reproduced at home. Thus, the company’s logic of “care for the environment” (in terms of protecting the immediate environment from the damaging consequences of oil and gas extraction, as well as encouraging an environmental ethic with their “Sustainable HomeWorks” program) is contradicted by encouraging higher GHG emissions as a symbol of higher status.

Giving activity spaces the labels “home” and “work” may hide deeper and more ambivalent understandings of the meanings of these places. When Harper is asked by the interviewer how he sees the relationship between his life offshore and onshore, he answers by simultaneously describing strong and permeable borders between his “separate lives.”

*But in terms of lives, (…) it might sound daft, but you’ve got a family at home and you’ve got a family offshore. Because these are the people that you’re living with 24 hours of the day, seven days a week, for two weeks! And you become pretty attached, quite emotional. You hear about their families*. *(…) – there’s similarities, and you establish close bonds. (...) While they are totally separate lives, because what happens at work stays at work, (….) I keep a lot of my emotions from work separate to emotions from home. Equally, there may be something going on in my home life and I try and keep it separate from my work life. But there’s times that there is an overlap. And over the years I’ve got very close to maybe just twelve colleagues and their families. And we meet up every year (…) And it’s really nice to pull it all together.*

Harper describes how, in spite of trying to keep both lives separate, practices, and meanings connected with family life at home are replicated at the workplace, while actual events together with the emotions they trigger are not transferred from one place to the other. The combination of spatial closeness, the sharing of tasks and daily life from dawn to dusk, create more intense experiences than those normally found at work and are therefore conducive to the reproduction of domestic practices at work and *vice versa*. That both lives are not as separate as Harper describes them, becomes clear in comparison to Dale’s description of “home from home.” While the latter talked about male bonding, Harper emphasizes the existence of “two families.” This reflects how the logics of home (in its broad sense) guide the perceptions of life at work. Other workers recounted how attempts are made to make the workplace more homely, especially at poignant moments in the year (e.g., Christmas): “*I’ve seen some fantastic creations. There was one year, (…) a fireplace appeared in the Control Room. So we had candles, carriage clock, and the flames in the grate and the stockings hanging off it – it was absolutely incredible!*” (Frank McKeen). For Robin Banks, such actions only served to highlight what they were missing: for “*… other guys it was just winding them up. Because like at the end of the day you’re on an oilrig.*” “Home from home” in the workplace might be seen, in Clark’s theory, as “blending,” where an effort is made to re-create the practices usually associated with the domain of home into the living quarters of the off-shore platform. The different reactions to these practices show the ambivalence of such blending as different ways of dealing with the absence of home. Despite his best efforts to keep his worlds apart, Harper cannot shed GlobalOil’s safety culture as he boards the helicopter back to the mainland:

…*my family, (…) they have a life without me, and (…) a different life when I am home, because I have different standards. While it’s nice to see each other, there can be a clash at times! It’s “Oh, you’re back again … We don’t bother with that when you’re away!*” *Like if I (…) do some gardening, I’ll wear safety boots, and I’ll put goggles on, and (…) ear-defenders. And my wife will go in the garden … in her stockinged feet and no gloves! And she’s doing the gardening, and [I will say] “Whoa, whoa, whoa! No, no, you need to!” “Oh, I’m okay! You get back offshore!*”

Harper’s actions are understandable in that he is concerned for his wife’s safety. But from the point of view of his wife, she is behaving appropriately. For her, Harper’s intervention constitutes what Mary Douglas refers to as a breach of the moral order as the “… moral component of assigning reality to different categories becomes particularly apparent when things get out of place” (Wuthnow et al., 2009, p. 87). Not only objects can be out of place but also behaviors and the logics guiding them. Given the essential logics we have laid out for corporations and families, the clash of practices here is a reversal of what we have claimed: the workplace practices signify care, while the home practices signify an ordered routine and efficiency put in place by Harper’s spouse. Harper’s wife does not experience his behavior as care but as an intrusion into her way of life, undermining her sense of control, her identification with her home, by rendering her practices as inferior. Harper’s descriptions of work and home demonstrate loyalty to both his workplace and his home, while his identity is shaped predominantly by his long work experiences and thus tends to create tensions at home rather than at work. While in Dale’s case, his shorter experiences at the workplace led him to use company logics as a legitimation to continue his environmentally damaging practices outside work, in Harper’s case, his long work experience led him to identify with environmental and safety practices at work to the point that he aimed to transform his home according to the safety rules guiding his workplace.

Taking safety practices from work to home was a story told by many of our informants as well as the resistance their partners posed to such a transference. Kia Alani, an offshore worker, relates.

...*when I first joined, I saw the strict rules about holding the handrail. You’re thinking, “Holding the handrail! Do you know how many people have touched the handrail? (…) And then just three days ago the lady sitting next to me, said, she always tells her husband (…): ‘Hold the handrail.’ and he always laughs and says, ‘Oh, please, it’s a GlobalOil rule!’ (…). And two days ago, he fell down from a flight of stairs in their house, all the way down, because he wasn’t holding the handrail!.”*

Kia Alani regarded this story as reflecting well on GlobalOil safety culture, as “*a lot of company policies and procedures to try and keep you safe*,” even when she had had another concept of safety concerning handrails.

Nick Stevens, at the time of the interviews had worked for GlobalOil for 37 years. He identifies with the company, which comes across as he talks about the safety record of GlobalOil, “*this is not just rhetoric; this is what I believe to be true – the safety of our employees, [the] health of our employees and also on environmental issues*.” In 2008, he “*… decided to change completely, to become … the Regional Discipline Advisor for Competence and also for the Skill Pool [Manager] and making sure that that was robust.*” Given this background, it is not surprising that he tended to treat the home like the oil platform – and *vice versa*: “*We had a great book [at GlobalOil], the A-Z of Safety it was called (…) So a bit like being offshore when I had my exercises, I used to – this is terrible, really! – I used to practise with my children, and press the alarm at maybe nine o’clock in the morning on Saturday; not too early – and they knew that they had to get up, get dressed, shout ‘Fire!’ and then go outside.*” Stevens was not blind to the incongruity of his actions in different settings. We can conclude that it is the “exercise” element of his practice, which in hindsight strikes him as “terrible.” Logics of discipline and compliance and the logics of care crash in this translation of work into home practices.

How can we explain that specifically safety practices were transferred from work to home? Shove (2010) argues, “… we need to understand how institutions, infrastructures, and daily life interact” (p. 1278). One management strategy which recognizes this is the concept of organizational culture (Schein, 2010; Schneider et al., 2011). Corporate culture can be defined as a contingent logic attributed to the organization. Safety was often discussed by our interviewees not as a set of rules and regulations, but rather as a habitual cultural driver. The Deepwater Horizon disaster (in 2010) featured in the narratives of only two workers, both explaining it as the management “cutting corners” and thus distancing their own company from it. Some of the most persuasive evidence for the effectiveness of organizational culture approaches comes from multi-national oil and gas corporations (Hudson, 2007). Building safety into everyday practices has been seen as essential to address high accident rates in an extremely hazardous working environment. To the degree that such practices become habitual, they are internalized and form part of people’s identities. Mike Wellwood provided an example of the processes through which this happens: “*And people will start meetings with a safety message, which will include examples from home. And you will see sometimes articles online about safety in the home as well as the workplace…*” For Stevens, the benefits of this led to GlobalOil playing a critical role in social change through the encouragement of border crossing: “*GlobalOil actually contributes to social change as well. Because what people learn at work they do take home with them and become more aware*.” The conflict he described when he “exercised” safety practices at home has disappeared in this statement. While practices are taken from work to home because they have become a part of people’s identities, there is simultaneously the need to strengthen the borders between both places by “forgetting” the conflicts these transferences can elicit.

A psychological interpretation of the desire to adopt or maintain similar practices on either side of the work/home border might be cognitive consistency (Thøgersen, 2004). A more persuasive driver for the adoption or maintenance of consistency means that similar types of practices may be pursued or avoided in each space. While taking behaviors home may result in cognitive and emotional consistency for the person who straddles the border, it simultaneously creates cognitive and emotional dissonance for the person remaining at home, since that behavior is not part of the accepted logic and assumptive world of *their* context.

The study of our two protagonists shows that it would be short sighted to talk about individuals either drawing borders strongly or permeating them. Kevin Dale and Andy Harper did both: they emphasized the differences between home and work but at the same time described their workplaces as another home into which they invested emotional attachment, thus carrying meanings, emotions, and identities from one place to the other. In terms of carrying practices across borders, though, both men were quite different. Dale engaged in similar (male culture) and contrasting behaviors at work and at home: being responsible for environmental protection at work, he was quite conscious of the significant carbon footprint he created in his leisure time without expressing any regrets. Dale reproduced his company’s double standard of environmental care and environmental destruction in his everyday life outside work subconsciously. What he took with him from work to home was not a specific practice but a tacit institutional logic.

Harper’s border crossings were in line with the logic of care at the workplace: care for his family’s safety when he urged his wife to wear protection gear in the garden. But these practices were seen by family members as “matter out of place,” as the intrusion of a work logic into the logic of the home. The institutional logic of care for safety had become a logic according to which Harper organized his personal life, but he could not carry this logic and the respective practices into his home where his family lived according to a different logic, which required other priorities of care.

In the following sections, we shift the focus of our analysis from a diachronic analysis of individuals’ home–work relationships to a synchronic analysis of border crossing practices.

## Synchronic Analysis: Instances of Border Crossing – Permeability, Strong Borders, and Blending

### Permeability

Paradoxically, when talking about sustainable environmental practices, the activity mentioned most frequently (as it would be by the population at large) was waste reduction, not energy conservation or carbon reduction. Robin Banks, a subcontractor whose father worked for another multinational oil corporation, was in his late 20s but had experience of working on numerous offshore platforms for a variety of companies. Separating and recycling waste is standard practice in most “good” companies which has served to reinforce the habit: “*I do a lot of recycling at home. I used to do it a bit before, but now, seeing all the segregation bins offshore, it’s encouraged me to do a lot more at home as well.*”

Jim Roberts worked at HQ in London. His role had been to promote sustainability in respect of GlobalOil’s real estate: “*…my main project has been on the carbon reduction commitment in the United States*.” He was skeptical about carbon reduction actions at home. If it did occur it was in his view, “*Because we’re in the CO_2_ business …people tend to know, therefore they tend to do things just because of their knowledge.*” Frank McKean commented: “*Do I take what I do at work home with me? I think so..., even the type of car that you drive. Looking at the CO_2_ emissions aspect of your car, (…).– (…). ‘Oh, how much carbon is it?.*”

All the information, persuasion, and education to encourage the generalization of actions is in vain if the conditions in which people live and work do not allow them to change. Enabling actions of government in providing an infrastructure that encourages change are critical: “*I am quite frustrated with my own home life (…) we could waste-segregate more and we could recycle more*.” This lack of recycling, McKeen reveals, is due to the Highland Regional Council not taking a larger range of recyclables.

### The Power of the Economy

Andy Harper was one of the two workers we interviewed who had made an effort to install solar panels on his house. The other was Steve Morris, Aberdeen born and bred, who had always engaged in energy-saving activities such as salvaging remnant insulation panels from his previous company and using them to insulate his ceilings and under-floor cavities. He bemoaned the reduction of the government’s feed-in tariff incentive (a subsidy for domestic renewable energy production which goes into the national grid and for which the householder is paid), and the absence of any financial support for the initial capital cost: “*If the government even gave you a grant to get it, or met you halfway or something, I’d probably jump at it.*”

Arguably, economic reasoning is as much a logic of home as it is part of the logic of profit at work. The question is one of priorities. One can imagine decisions at home, where quality or well-being take priority over economic reasoning. Thus, prioritizing saving money over environmental protection can be seen as a way in which the corporate logic of cost efficiency seeps into the home domain and prevents significant environmental practices.

At the time of this research, GlobalOil launched an “in-house” campaign “Sustainable HomeWorks” to encourage the workforce to act more environmentally sustainably including reducing their carbon emissions at work and home. There is, of course, an irony about one of the world’s largest oil and gas producers and carbon emitters encouraging its staff to act more sustainably. What these practices taken from home to work show is that there is a need for “interinstitutional relationships” (Thornton et al., 2012), e.g., for a government infrastructure which allows people to transfer practices from one place to another.

### Strengthening the Border: Resistance and Compensation

When asked about a poster in the offices which warned about accidents in the home, Luc Vermeeren, an Onshore Project Manager in his early 40s, said, “*I mean, you don’t want the company to fully start determining your home life as well.*” One can trace Luc Vermeeren’s rejection of border crossing to his experiences as a young man. From the age of 17 or 18, he was a member of *Loesje*,^4^ a Dutch political organization which raises public awareness by putting up posters on issues such as the environment and racism. He took a year of unpaid leave at one point and traveled to Latin America and South-East Asia with a friend. His friend intended to go into Aid work, but Luc became frustrated by seeing how people lived, arguing that “*…they didn’t have this drive to try to make the best out of things they could. I think we said, “Well, forget it, (..). I’ll just leave it as it is, because there’s no use trying to push people into a direction they don’t want to be pushed.*” His resistance to the company’s intervention into life at home can be regarded as a principle acquired through life experiences before he entered his present workplace. He is an example of how identities acquired outside work can constitute a resistance to the logics of the workplace.

Kia Alani, a chemical engineer in her late 20s has worked for GlobalOil both off- and onshore. Thinking back to her time offshore, Alani described the practice of recycling: “*So you have like the cans, the bottles, you have like paper. …… and people are encouraged to do that as well.*” But then, guilty, she said “*Phhh! Don’t know if I should be saying this, though, … I do, at work. But when I go home, I just put everything in one, and that’s it! [laughs] Yeah, sorry, I know!*’ But there are other practices, she does take home: *‘...what they try and encourage us to do is switch off your monitors, (…). … which I now apply at home, be it my laptop, light bulbs in my room, (…) – with the TV as well. So that I do take home!*”

This sheds some light on the conditions under which people carry practices from work to home. Switching off electrical appliances carries more weight than recycling probably because it implies saving energy, and thus money. By contrast, recycling as at work is a lot of effort. It might be an act of quiet resistance or simply of compensation to “*put everything in one*.”

### Blending – Home as a Transitional Border

Our last example demonstrates that the relationships we are dealing with enable individuals to act as carriers of practices between institutions where these meet in the home. Frank McKeen introduced his partner to Six Sigma (Pande et al., 2000), a set of techniques developed for improving industrial processes and reducing defects. It had been adopted by GlobalOil in order to reduce waste and improve efficiencies with, he claimed, “huge effect.” He took the ideas home to his partner who took them to her boss, who then applied them to his business. In the reverse direction, having learnt from his spouse about the “Kaizen” management technique (Recht and Wilderom, 1998), he realized how GlobalOil could make its waste processes more efficient: “*So they had a recycle route for high-density plastics, and I knew that on the Kittywake we were using these drums and they were just going to landfill. But here was this readymade disposable route, so we joined up that two aspects of it...*” Company-to-company border crossing is not new, but this example is significant because the transfer is mediated through domestic conversations. Frank McKeen was one of the workers who enjoyed the workplace as a “home from home.” In turn, he did not shy away from converting the kitchen table into a workplace, where he and his partner assumed the role of managers thinking about how to improve their company’s effectiveness, blending work and home. Workplace logics materialize at home, the kitchen table becomes a space of creative innovation where two workers internalize the essential logics of their respective employers and help their production processes.

## Concluding Comments

In our initial research, industrial workers told us stories about how they took practices related to a “safety culture” at work home and the effect this had on their families and friends. Safety practices are more significant at work, because individuals and corporations receive more immediate feedback from health and safety incidents than from climate change (Gifford, 2011). In the case of industrial accidents, the reputational costs as well as financial penalties^5^ tend to fall on the company, while climate change is still regarded as a negative externality. Consequently, we realized that if we explored the relationship between the two domains and the ways in which individuals transition from one place to the other more generally we could also get a better understanding of how *environmental* practices, meanings, and identities might become generalized through transitions from work to home and *vice versa*. We therefore decided not to reduce our analysis to the few instances where people talked about environmentally relevant practices, but to include other practices that were transferred from one domain to the other. Theoretically, this was important as it set workers’ practices in the context of how they made sense of their relationship between the places of working life and the places of home life.

If practices were taken from one place to another, it was usually from work to the home. This indicates the power that company logics have in relation to the more malleable family logics. Borders were especially *permeable* between work and home when practices at work were homologous to those at home. The emphasis of the company on such environmental practices is ironic given the fact that the oil industry is a key producer and driver of GHG emissions. Knowledge about the dangers of GHG that comes with working in the oil industry also led to more significant pro-environmental practices like installing solar panels on the house by a few workers. But the internalized logics of the company served to reinforce practices only if they were seen to be economically efficient. When practices learned at the workplace are carried home, family members may *strengthen* border controls, because they experience these practices as an intrusion into a place they value and control. Some workers’ resistance to company demands of taking work practices home were a result of bringing logics acquired in their life course to bear on their relations to the company.

Another phenomenon was *blending* when subjective values individuals give to their home were taken into the workplace making it a “home from home.” Blending also occurs when family relationships at home draw on the logic of the corporation such that beneficial production practices are communicated between companies through the home.

Clark’s theory of border crossing places the individual as a purposeful agent at the center of managing the relations between workplace and home. We argue that the theory gains strength by combining it with a revised version of an institutional logics perspective. Recognizing that subjective values given to different places are connected to the structural, societal logics of those places, provides a framework of opportunities, constraints, and priorities for action. This enables us to understand better why some people under specific conditions draw strong borders between places, while under other conditions the same people experience borders as permeable, allowing the flow of practices, which may then operate “out of place.” We found Douglas’s (1966) ideas of moral ordering within a social setting also to be useful in understanding the meanings of home and work and how they may collide. We found that if we are to understand whether and how practices, meanings and identities are taken from one place to another then we need to analyze the process of border crossing on a number of levels while differentiating between different kinds of institutional logics.

Differentiating between essential and contingent logics has enabled us to show how the former dominate the latter. In our case, the contingent logic of care for the environment stood in contrast to the logic of producing a profitable product and played therefore a subordinate role in the everyday working life, mirrored by the marginal role it played in the accounts of workers. By contrast, the contingent logic of care for the safety of employees was connected to the essential logic of the company (since the costs for injuries and accidents are its responsibility) and could therefore become part of the company culture. As a result, safety considerations became part of workers’ identities, creating an attachment to their workplace and motivating them to carry the respective practices from work to home.

How individuals manage the borders between work and home depends not only on their position in the different domains as Clark argues, but also on the ways in which they make sense of their life trajectories and develop their identities. Kevin Dale a young worker with a love for motorbikes, cars, and frequent traveling is aware of his carbon footprint but does not present this as a problem, subconsciously reproducing the tacit logic of his company which fosters a logic of environmental care, but needs to follow the logic of profit thus contributing to environmental destruction through its production process and its product.

By contrast, Andy Harper, whose account centered around experiences of environmental protection in his family history and who has witnessed the development of a safety culture during his long years in the oil industry has developed an identity that leads him to carry work practices home, even if this produces conflicts: simultaneously he consciously aims to keep his “two families” at work and at home apart. Not only logics and practices travel but also emotions. One can argue that the essential logics of home – care, support, and emotional closeness need to cross the borders into the workplace. We found that this becomes especially clear when analyzing an extreme case like work on an oil platform, where the two domains differ decisively and there are larger time lapses between a presence at home. This is compounded when individuals may feel vulnerable since they are working in a dangerous environment where mutual support is essential.

Our analysis leads us to four key conclusions concerning border crossing between the place of home and the place of work. First, the institutional logics of home and work will influence the individuals acting in these places. Second, the way in which this happens has to be analyzed in each of the different domains. On the side of the subject the domains of practices, meanings, and identities can differ in terms of how institutional logics are carried across borders or not. Third, the transfer (or not) of practices, meanings, identities and logics needs to be analyzed as a process which happens consciously as well as subconsciously. Fourth, in order to understand this process and its complexity in its different domains, we need to analyze the respective institutional logics of the places between which the process of border crossing takes place as well as the life-trajectories of individuals as purposeful actors of this process. As we found, for example, in the case of Kevin Dale, an individual may regard the same border as both strong and permeable depending on whether they perceive the border rationally or emotionally, for instance. Our case studies showed that carrying practices across the work-home divide involves contextual meaning-making, deliberation, conflict, negotiation, and decision-making. As noted (Kossek and Lautsch, 2012), many organizational studies tend to privilege either individual or organizational factors in boundary management. As with their study, we argue for an understanding of individuals’ work/home relationship as being “nested” within organizational logics. However, our study does not take a role-based approach or center on boundary management but sees the logics of institutions as being a critical factor in understanding the creation and management of boundaries.

If it is deemed desirable that safety and environmental practices should cross the home/work divide, then it has to be appreciated that the process will not happen by osmosis but has to be planned and facilitated. This requires companies to recognize the elements occurring in the process: deliberation, conflict, negotiation, decision-making, and power relations. What are the conditions under which people make decisions that increase or decrease the probability of the transfer of pro-environmental or other behaviors across different institutions with different logics? The logic of care can be present in the workplace through “family-friendly” policies and facilities (e.g., crèche facilities), but also through safety regulations and a “health and safety culture.” The latter tend to be limited to reducing the immediate threat of the production process to the health and safety of workers and the surrounding environment (or as one interviewee said they often referred to the sea as – “the big blue skip”). Health and safety is not regarded as a negative externality in the way that the environment is. Treating environmental impacts as internalities might lead to both the development of an environmental culture akin to the safety culture, and a longer term appreciation of the cost of oil production. We would suggest that future research into the relationship between work and home could benefit from analyzing not only the process of carrying practices across the work/home border but also the multiple levels of essential and contingent logics that guide practices, meanings, and identities in each domain as well as the life trajectories of individuals as the actors of border crossing.

We have seen an opportunity in this paper to provide a permeable border between environmental psychology writings on place-related environmental behaviors and sociological writings on societal and institutional factors influencing decision-making. While we would agree that place-meaning can be an important condition influencing people’s pro-environmental attitudes and behaviors, we would also argue that those places where place is salient such as the home and the workplace are also institutional settings which are subject to particular logics. When we, as environmental psychologists, talk of the importance of context we have not always been particularly specific in articulating precisely what this context is. We would have little difficulty in agreeing that it includes social relations and the physical environment. But it also includes society’s institutional structures with their attendant logics. We suggest that the contribution of this paper to the research literature on the transfer of pro-environmental behaviors and practices across places is that it argues for the need for researchers to attend to the institutional logics which are no less part of the context which drives our environmental attitudes and behaviors than other structural or processual considerations.

## Author Contributions

DU and NR undertook the analysis and interpretation of the interviews in equal measure. DU and NR co-wrote the final text.

## Conflict of Interest Statement

The authors declare that the research was conducted in the absence of any commercial or financial relationships that could be construed as a potential conflict of interest.

## Footnotes

1 We have changed the name of the oil and gas corporation, individuals, and oil fields to ensure anonymity obligations.

2 These numbers reflect the gendered division of work. We do not have the space to conduct a gender specific analysis of the material.

3 All subjects gave verbal and informed consent to interview and use the interviews for our analyses. The research team followed the British Psychological Society BPS Code of Ethics and Conduct (2009) which states that “*The way in which consent is sought from people to participate in or otherwise contribute data for research should be appropriate to the research topic and design, and to the ultimate outputs and uses of the analyses. It should recognise in particular the wide variety of data types, collection and analysis methods, and the range of people’s possible responses and sensitivities. The principle of proportionality should apply, such that the procedures for consent are proportional to the nature of participation and the risks involved.*” None of the interviewees were children, vulnerable adults, or adults with severe physical or mental impairments, and thus given the subject of the research it was felt that verbal consent was proportional. All interviewees volunteered to be interviewed and were informed, *inter alia*, of the following: the aim(s) of the project; the type of data to be collected; the method of collecting data; confidentiality and anonymity of both the interviewee and the company they worked for; the right to decline to offer any particular information requested; the opportunity to withdraw from the study at any time with no adverse consequences; and how the data will be used and planned outcomes.

4 http://www.loesje.org

5 The Piper Alpha explosion (1988) in the North Sea resulted in 167 dead, and an insured loss of *£*1.7bn (Woolfson et al., 1996). BP has estimated the final cost of the Deepwater Horizon blowout in the Gulf of Mexico to be $62bn (BP, 2016).
